# Palatal Injection does not Block the Superior Alveolar Nerve Trunks: Correcting an Error Regarding the Innervation of the Maxillary Teeth

**DOI:** 10.7759/cureus.2120

**Published:** 2018-01-28

**Authors:** Joe Iwanaga, R. Shane Tubbs

**Affiliations:** 1 Seattle Science Foundation; 2 Neurosurgery, Seattle Science Foundation

**Keywords:** anatomy, innervation, local anesthesia, maxillary nerve, nerve block, tooth

## Abstract

The superior alveolar nerves course lateral to the maxillary sinus and the greater palatine nerve travels through the hard palate. This difficult three-dimensional anatomy has led some dentists and oral surgeons to a critical misunderstanding in developing the anterior and middle superior alveolar (AMSA) nerve block and the palatal approach anterior superior alveolar (P-ASA) nerve block. In this review, the anatomy of the posterior, middle and anterior superior alveolar nerves, greater palatine nerve, and nasopalatine nerve are revisited in order to clarify the anatomy of these blocks so that the perpetuated anatomical misunderstanding is rectified. We conclude that the AMSA and P-ASA nerve blockades, as currently described, are not based on accurate anatomy.

## Introduction and background

Anesthetic blockade of the posterior superior alveolar (PSA) branch of the maxillary nerve has played an important role in the endodontic treatment of irreversible acute pulpitis of the upper molar teeth except for the mesiobuccal root of the first molar tooth [1, 2]. This procedure requires precise anatomical knowledge of the pterygopalatine fossa and related structures in order to avoid unnecessary complications and to make the blockade most effective.

The infraorbital nerve gives rise to middle superior alveolar (MSA) and anterior superior alveolar (ASA) branches. However, blockade of the greater palatine nerve (GPN) has been used, especially for periodontal treatment, to anesthetize the palatal mucosa, including the posterior part of the hard palate and its overlying soft tissues, anteriorly as far as the first premolar and medially to the midline, and palatal gingiva.

The superior alveolar nerves course lateral to the maxillary sinus and the GPN travels through the hard palate [1, 2]. However, regarding nerve blockade, the difficulty in understanding its three-dimensional anatomy has led some dentists and oral surgeons to develop two anesthetic blockades that are based on erroneous morphology. These blockades are known as the anterior and middle superior alveolar (AMSA) nerve block and palatal approach anterior superior alveolar (P-ASA) nerve block. Recently, schematic drawings and photographs of the AMSA nerve block have been shown in several reports [3, 4]. However, some of these have incorrectly depicted the palatal gingiva and mucosa as being innervated by the ASA and MSA branches of the infraorbital nerve and the upper teeth by the GPN. In addition, these have also shown the incisive canal as being supplied by the ASA.

With such a clinically significant error being propagated in the dental literature, we aimed to review this anatomy and investigate the origin of the anatomical misunderstanding. With an improved understanding of the anatomy of the innervation of the maxillary teeth, unnecessary anesthetic blockade will be avoided with resultant improved patient outcomes.

## Review

Anatomy of the superior alveolar nerves (posterior, middle, and anterior branches)

The upper teeth are supplied by three superior alveolar nerves that arise from the maxillary nerve in the pterygopalatine fossa or infraorbital canal. The PSA branch leaves the maxillary nerve in the pterygopalatine fossa and runs anteroinferiorly to enter the posterior alveolar foramen on the infratemporal surface of the maxilla (the posterior wall of the maxillary sinus). It then descends under the mucosa of the maxillary sinus (or through the bony wall of the maxillary sinus). Finally, this nerve divides into small branches that unite as the molar part of the superior dental plexus, which supplies the ipsilateral molar teeth, gingivae and the adjoining part of the cheek [1]. As it runs in the infraorbital canal, the MSA branch of the infraorbital nerve runs downward and forward in the lateral wall of the maxillary sinus. Distally, it terminates as small branches that join the superior dental plexus, which supplies small rami to the upper premolar teeth. The MSA branch is variable and may be duplicated or absent. The ASA branch arises from the lateral side of the infraorbital nerve near approximately the midpoint of the infraorbital canal. It travels under the infraorbital foramen through the bone to pass medially toward the nose and finally turns downward and divides into branches that supply the incisor and canine teeth. It also contributes to the formation of the superior dental plexus [1].

Anatomy of the greater palatine and nasopalatine nerves

The GPN is one of the branches of the maxillary nerve that enters the greater palatine foramen to travel within the oral cavity along the roof of the mouth. It travels downward and forward giving rise to numerous branches to the ipsilateral palatal mucosa, gingiva, and glands of the hard palate as it approaches the incisor teeth. The GPN communicates with the terminal branch of the nasopalatine nerve.

The nasopalatine nerve leaves the pterygopalatine fossa through the sphenopalatine foramen to enter the nasal cavity. It passes across the cavity to the back of the nasal septum, runs downward and forward through the nasal septum in a groove in the vomer and then turns down through the incisive canal to traverse the incisive foramen at the anterior part of the hard palate. It supplies the lower part of the nasal septum and the anterior part of the hard palate where it communicates with the GPN.

Propagated misunderstanding of the AMSA nerve block

The technique of AMSA nerve block was first developed by Friedman and Hochman based on the description in the old literature that pulpal anesthesia can be accomplished from a palatal injection as documented by Fischer [5] in 1911 and Nevin [6] in 1927. The concept of the AMSA nerve block was conceptualized as “the ideal maxillary injection would produce a rapid onset of profound pulpal anesthesia for multiple teeth from a single needle penetration on palatal mucosa.” Although some literature has mentioned success with this technique [7], most studies have only compared the traditional syringe technique with the computed controlled delivery system [8].

**Figure 1:**
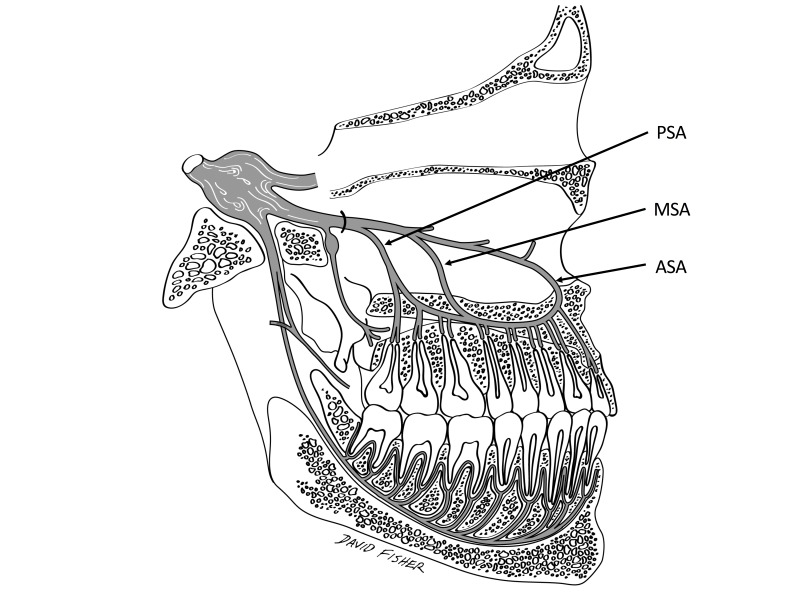
The PSA, MSA and ASA as depicted by Friedman and Hochman (1998) as if they innervate the teeth through the hard palate. The ASA is drawn as if it is the terminal branch of the infraorbital nerve. ASA: Anterior superior alveolar branch of the infraorbital nerve; MSA: Middle superior alveolar branch of the infraorbital nerve; PSA: Posterior superior alveolar branch of the maxillary nerve.

Friedman and Hochman [9] illustrated the AMSA nerve block although this depiction was incorrect (Figure 1).

In their drawing, the infraorbital nerve or maxillary nerve is shown as giving rise to branches to all of the maxillary teeth through the hard palate as if they traveled in a similar course as the GPN. Furthermore, in their drawing, the main trunk of the infraorbital nerve becomes the ASA as if it were the terminal branch of the infraorbital nerve. The course of the ASA in this figure might be confused with the pathway of the nasopalatine nerve toward the incisive foramen through the nasal septum. Therefore, this publication has added to the confusion of the course of the superior alveolar branches, GPN and nasopalatine nerve, and has probably resulted in patients undergoing unnecessary nerve blockade.

Concept of the P-ASA nerve block and misunderstanding

**Figure 2:**
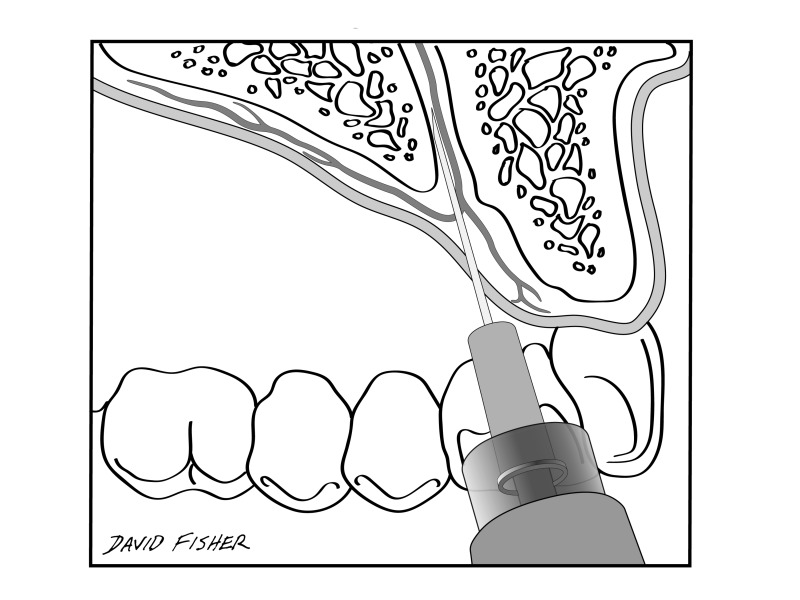
The proposed drawing of the P-ASA by Friedman and Hochman (1999). P-ASA: Palatal approach anterior superior alveolar.

The P-ASA nerve block was proposed by Friedman and Hochman in 1999 [10] as a primary method to achieve bilateral pulpal anesthesia of the six anterior maxillary teeth. The objective of this injection is to put the needle at the entrance of the incisive canal and maintain contact with the inner bony wall with a depth of approximately 6 to 10 mm (Figure 2).

According to Friedman and Hochman [11], the use of the P-ASA nerve block theoretically would be a great advantage because only one injection would anesthetize all the anterior teeth bilaterally [10]. The P-ASA nerve block has also been useful in cosmetic and pediatric dentistry because this procedure does not result in numbness of the upper lip [11]. For esthetic restorative dentistry, when anterior esthetic restorative procedures are being performed, using a P-ASA nerve block appears to be useful [11]. For pediatric dentistry, it is said that this is an ideal procedure because the patients do not scratch at the upper lip after injection [12]. However, the P-ASA nerve block is anesthesia aimed at the incisive canal (nasopalatine nerve). As the ASA branch courses away from the incisive canal, it is not logical to call this the palatal approach “anterior superior alveolar” nerve block. In addition, most research has focused on outcomes of the P-ASA nerve block with a computed controlled delivery system and not on the P-ASA nerve block itself [13, 14].

Application to periodontal treatment

Several studies have reported the effectiveness of AMSA nerve block in periodontal surgery and scaling and root planing (SRP) [3, 15-17]. However, the AMSA nerve block could anesthetize the palatal gingiva and periodontal ligament because its injection site corresponds to the course of the branches of the GPN which means the anesthetic solution is carried by the branches of the GPN and not by the AMSA. The blanching of the buccal gingiva could be achieved by diffusing the anesthetics into the buccal gingiva which would result in anesthesia during SRP [4]. The blanching of the buccal gingiva often occurs after infiltration anesthesia to the palatal gingiva so there is no evidence for the success of the AMSA nerve blockade.

Criticisms

Corbett, et al. [18] reported that the infraorbital nerve (ION) blockade is more effective for the canine and premolar teeth compared to the AMSA nerve blockade. Although the AMSA nerve blockade was more successful than the ION blockade in attaining incisor anesthesia, it was ineffective for anesthesia of the central incisors, as assessed by rigorous electronic pulp testing. According to Velasco and Soto [19], the AMSA nerve blockade, using a conventional syringe, obtained a 66% success rate for the second premolar, 40% for the first premolar, 60% for the canine, 23.3% for the lateral incisor, and 16.7% for the central incisor. These authors concluded that as a first line treatment, the AMSA nerve blockade is disadvantageous for clinical application based on its unpredictable anesthetic success.

**Figure 3:**
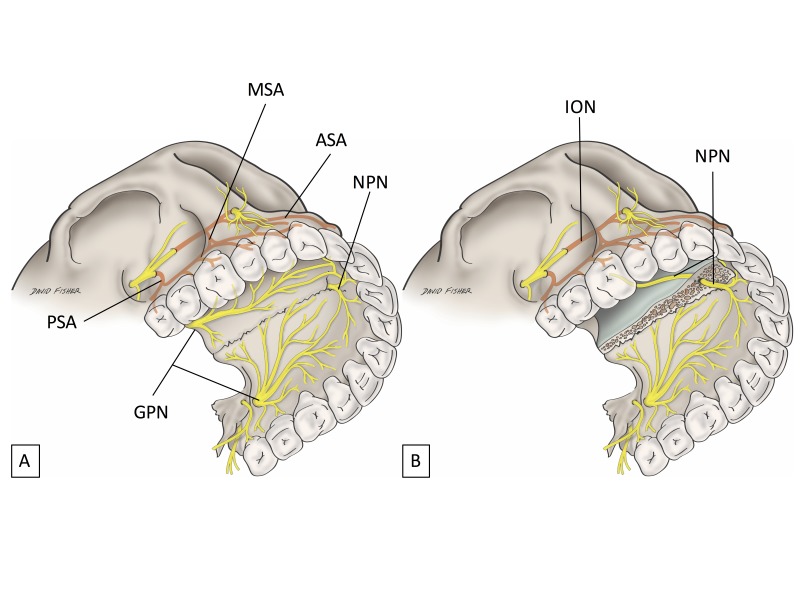
Neither AMSA nor P-ASA nerve blockade is close to the main nerve trunk of the superior alveolar nerves. (A) Relationship of the superior alveolar nerves and GPN. (B) Relationship of the superior alveolar nerves and NPN. AMSA: Anterior and middle superior alveolar; ASA: Anterior superior alveolar branch of the infraorbital nerve; GPN: Greater palatine nerve; ION: Infraorbital nerve; MSA: Middle superior alveolar branch of the infraorbital nerve; NPN: Nasopalatine nerve; PSA: Posterior superior alveolar branch of the maxillary nerve.

Burns, et al. [20] reported a prospective, randomized, double-blinded study to evaluate the P-ASA nerve block using computer-assisted injection and this resulted in successful anesthesia in only 32 to 58% of patients. These outcomes, in terms of anatomy, make sense. A nerve block is defined as a local anesthetic solution placed near a main nerve trunk [21]. Neither AMSA nor P-ASA nerve blockade is near the main nerve trunk of the superior alveolar nerves (Figure 3).

Tiny branches could exist between the GPN and AMSA branches and between the nasopalatine nerve and the ASA branch. However, if we revisit the anatomy of the maxilla, palate and nasal septum, it is easy to understand how many reports in the literature have made critical mistakes regarding AMSA and P-ASA nerve blockade.

## Conclusions

The AMSA and P-ASA nerve blockades are not based on accurate anatomy. The term “AMSA” nerve blockade is infiltration anesthesia to the root of the anterior, canine and premolar teeth, and blockade of the branch of the GPN. This anesthesia might provide numbness to some extent, but this comes most likely from “infiltration anesthesia.” The term “P-ASA” nerve block is similar to the nasopalatine nerve block. The innervation of the maxillary teeth should be revisited in order to provide the best local anesthesia to patients without unnecessary injections and the potential for associated complications.
